# IRF9 and XAF1 as Diagnostic Markers of Primary Sjogren Syndrome

**DOI:** 10.1155/2022/1867321

**Published:** 2022-09-12

**Authors:** Lu Xiao, Zhou Yang, Shudian Lin

**Affiliations:** Department of Rheumatology, Hainan General Hospital (Hainan Affiliated Hospital of Hainan Medical University), Hainan 570311, China

## Abstract

**Objective:**

Primary Sjogren syndrome (pSS) is characterized by lymphocytic infiltration of the salivary and lacrimal glands. It is a chronic systemic autoimmune disease. Genetic contributions and disturbed biological systems are the two major causes of pSS, but its etiology is unclear. This study is aimed at identifying potential pSS diagnostic markers and mechanisms at the transcriptome level.

**Methods:**

Whole blood datasets of patients with pSS were downloaded from the Gene Expression Omnibus (GEO) database. Differentially expressed genes (DEGs) were identified using the online tool, GEO2R. R software was used to perform enrichment analyses to understand the functions and enriched pathways of the DEGs. A protein–protein interaction network was constructed to identify hub genes and significant gene clusters. The least absolute shrinkage and selection operator logistic regression was used to screen pSS diagnostic markers. The expression level and diagnostic performance of the identified genes were tested using another GEO dataset.

**Results:**

A total of 221 DEGs were screened from the whole blood samples of 161 patients with pSS and 59 healthy controls. Functional enrichment analysis demonstrated that DEGs were mostly enriched in defense response to virus, response to virus, and type I interferon signaling pathway. Cytoscape identified 10 hub genes and two gene clusters. IRF9 (AUC = 0.799) and XAF1 (AUC = 0.792) were identified as pSS diagnostic markers. The expression levels of the two identified genes were validated by GSE51092.

**Conclusion:**

IRF9 and XAF1 were identified as diagnostic markers. The potential underlying molecular mechanism of pSS was explored.

## 1. Introduction

Primary Sjogren syndrome (pSS) is characterized by lymphocytic infiltration of the salivary and lacrimal glands. It is a chronic systemic autoimmune disease that is overwhelmingly dominated by dry mouth (xerostomia), dry eyes (keratoconjunctivitis sicca), and autoantibody production. It may be accompanied by multiorgan systemic manifestations, such as pulmonary fibrosis and B cell lymphoma [1]. The prevalence of pSS in the general population is 0.33%–0.77%. Its occurrence rate is 10 times higher in females than in males [2]. The mortality rate is high among patients with cryoglobulinemic vasculitis, parotid enlargement, and lymphoma [3]. Genetic contributions and disturbed biological systems are the two major causes of pSS, but the etiology of pSS remains unclear.

Differentially expressed genes (DEGs) among different groups of people can be discovered with the rapid progress of microarray techniques. In the past few years, several bioinformatic studies have revealed potential biomarkers in patients with pSS, which helped better elucidate the biological mechanisms of the disease. Chen et al. demonstrated that the expression levels of STAT1, MNDA, IL10RA, and CCR1 in the minor labial gland biopsy of pSS can serve as potential biological indicators for the disease [4]. Another study reported that PTPRC, CD86, and LCP2 are hub genes in the gland tissues of patients with pSS [5]. Li et al. identified seven key genes, namely, MS4A1, CD19, TCL1A, CCL19, CXCL9, CD3G, and CD3D, in gland tissues that have potential values for evaluating pSS severity [6]. Most past studies focused on gland tissues, but few studies focused on whole blood. In 2019, Yao et al. applied weighted gene coexpression network analysis to identify the potential pathways and hub genes that may be involved in pSS development [7]. Moreover, gene coexpression modules and hub genes in peripheral blood and parotid tissue associated with B cell levels in pSS were identified by Lei and Zhang [8]. The substantial pathogenesis-related processes of pSS have been studied, but researchers still need to elucidate the exact pathogenesis and the key pathogenic factor of the disease. Useful algorithms and machine learning methods help with the easy discovery of diagnostic markers to better understand disease pathogenesis [9]. Wang et al. established the value of information feedback models and proved that hybrid adaptive differential evolution algorithm is a good state-of-the-art algorithm [10]. Therefore, the potential key genes and pathway networks related to pSS development can be explored through the combination of microarray and bioinformatic algorithm. Whole blood sample is easier to obtain than gland tissues; hence, studying the hub genes that may have diagnostic and treatment value from whole blood is of great importance for better patient outcomes.

In this study, pSS-related sequencing data from a public database were downloaded to discover the potential diagnostic markers and biological functions of the disease. The microarray datasets for pSS and HC, namely, GSE84844 (GPL570) and GSE66795 (GPL10558), respectively, were downloaded from the Gene Expression Omnibus (GEO) database. Then, the data were integrated and reanalyzed. A total of 221 common DEGs were identified between pSS and healthy control (HC). DEGs were clustered with functional enrichment analysis and gene set enrichment analysis (GSEA). Furthermore, a protein–protein interaction (PPI) network was constructed using the online tool, Search Tool for the Retrieval of Interacting Genes/Proteins (STRING). Genes identified by the least absolute shrinkage and selection operator (LASSO) logistic regression and Cytohubba, a Cytoscape plugin to detect hub genes, were combined for further analysis. Subsequently, the two identified hub genes were validated using GEO dataset GSE51092 (GPL6884). The diagnostic accuracy of the identified hub genes for pSS was evaluated with the area under the receiver operating characteristic curve (AUC).

## 2. Materials and Methods

### 2.1. Data Download and Preprocessing

“Primary Sjogren syndrome” was used as the search keyword for the expression profiles of pSS in the GEO database [11]. Datasets containing the sequencing information of whole blood from patients with pSS and HCs were included. Finally, two datasets, namely, GSE84844 (GPL570) and GSE66795 (GPL10558), were selected as the test sets [12, 13]. Dataset GSE51092 (GPL6884), which includes samples from 190 patients with pSS and 32 HCs, was selected as the validation set [14]. Basic information, including platform, samples, source tissue, and attribution, is listed in Table 1. The overall flowchart is shown in Supplementary Figure 1.

### 2.2. DEG Screening

Online web-based tool GEO2R was applied to discover the DEGs between pSS and HC. Adjusted *P* < 0.01 was regarded as statistically significant. The overlapping DEGs from the two datasets were detected by the online tool Draw Venn Diagram (http://bioinformatics.psb.ugent.be/webtools/Venn/).

### 2.3. Functional Enrichment Analysis

Gene Ontology (GO) and Kyoto Encyclopedia of Genes and Genomes (KEGG) pathway analyses of the identified DEGs were performed by R packages (clusterProfiler, ggplot2, and GOplot) [15]. clusterProfiler was applied to analyze the DEGs. ggplot2 and GOplot were used to visualize the results.

### 2.4. GSEA

GSEA was conducted for all genes from the two gene datasets (GSE84844 and GSE66795) using R studio. ggplot2 was used to visualize the results. The gene set arrangement was performed 1000 times per analysis. Gene sets were considered significantly enriched when false discovery rate (FDR) < 0.25, adjusted *P* < 0.05, and ∣normalized enrichment score (NES) | >1.

### 2.5. PPI Network Construction

A PPI network was constructed by the online tool STRING [16]. The cutoff standard was set as a combined score of >0.4 [17]. Then, the results were visualized by Cytoscape software. Molecular Complex Detection (MCODE, V1.5.1), a Cytoscape plugin, was used to identify significant modules (MCODE score ≥ 4) [18]. Moreover, the hub genes were chosen by Cytohubba based on a high number of associations with other genes in the PPI network [19].

### 2.6. Screening and Verification of Diagnostic Markers

LASSO logistic regression was used to perform feature selection to identify diagnostic markers for pSS. The expression matrix of the GSE66795 dataset was subjected to LASSO logistic regression. The LASSO regression analyses were conducted using the glmnet R package [20]. The genes from LASSO regression analysis and the hub genes identified by Cytohubba were combined for further analysis.

### 2.7. Statistical Analysis

RStudio was used to perform the statistical analysis. GSE51092 was used to validate the expression levels of the identified diagnostic markers. Wilcoxon rank sum test was used as the statistical method when the data were not normally distributed. Independent sample *t*-test was used when the data were normally distributed. Moreover, AUC, which was used to represent the diagnostic performance of the identified genes, was determined by RStudio. GLM function was used to build the logistic regression model. PROC package was used to analyze the receiver operating characteristic (ROC) curves. ggplot2 package was used to visualize the results. *P* < 0.05 was considered significant.

## 3. Results

### 3.1. Data Preprocessing and DEG Screening

**Figure 1:**
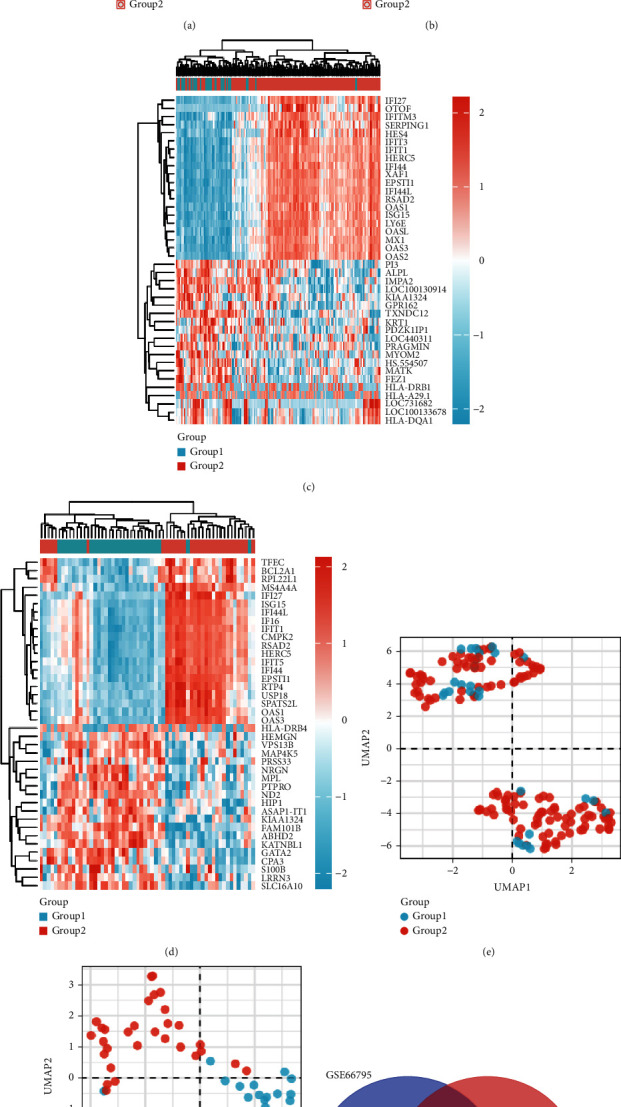
Identification of DEGs. (a) Principal component analysis (PCA) plot generated from DEGs in GSE66795. (b) PCA plot generated from DEGs in GSE84844. (c) Heat map of GSE66795. (d) Heat map of GSE84844. (e) Uniform manifold approximation and projection (UMAP) showing distinct clusters of DEGs in GSE66795. (f) UMAP showing distinct clusters of DEGs in GSE84844. (g) Venn diagram of common DEGs from the two datasets. Data points in red represent upregulated genes, and those in blue represent downregulated genes. Group 1 indicates HC and group 2 indicates pSS.

The overall workflow of the study is shown in Supplementary Figure 1. Two public pSS datasets, namely, GSE66795 and GSE84844, were downloaded to investigate the common DEGs involved in pSS. The principal component analysis (PCA) clusters for GSE66795 and GSE84844 are presented in Figures 1(a) and 1(b), respectively. PCA demonstrated that almost all variations were represented by HC and pSS in GSE66795 (16.1% and 10.1%, respectively) and GSE84844 (21.9% and 8.1%, respectively). In addition, Figures 1(c) and 1(d) show the heat maps and Figures 1(e) and 1(f) present the uniform manifold approximation and projection (UMAP) of the two gene datasets. Two clusters could be identified by UMAP analysis in GSE84844. As shown in Figure 1(g), 486 and 5466 DEGs were found in GSE66795 and GSE84844, respectively. After these, DEGs were integrated, and 221 common DEGs were discovered (Figure 1(g)).

### 3.2. Biological Functions of Common DEGs

**Figure 2:**
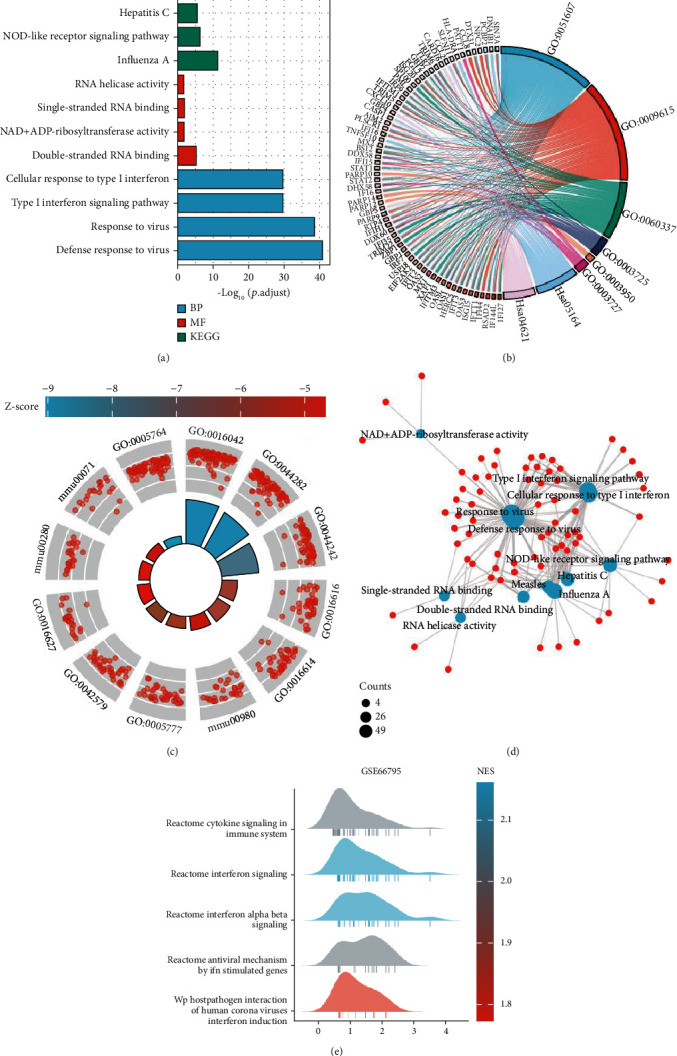
Functional enrichment of DEGs. Gene Ontology (GO) and Kyoto Encyclopedia of Genes and Genomes (KEGG) pathway analyses of DEGs. (a) Histogram, where the horizontal axis represents the number of DEGs under the GO or KEGG term. (b, c) Chordal graph, enrichment analysis combined logFC. On the basis of enrichment analysis, logFC of the molecule provided is used to calculate the corresponding *Z* score of each item and preliminarily determine whether the corresponding item is positively regulated (*Z* score is positive) or negatively regulated (*Z* score is negative). (d) Visual network of GO enrichment and KEGG pathway analysis of the overlapping DEGs. (e) Gene set enrichment analysis of GSE6675.

GO, KEGG, and GSEA were used to analyze the 221 common DEGs (Figure 2, Tables 2 and 3). Based on GO enrichment, the biological process acted on defense response to virus, response to virus, and type I interferon signaling pathway. Regarding molecular functions, these proteins played roles in double-stranded RNA binding, NAD+ ADP-ribosyltransferase activity, and single-stranded RNA binding. According to the KEGG pathway analysis, these proteins were involved in influenza A and NOD-like receptor signaling pathway (Figures 2(a)–2(d)). GSEA is designed to analyze the association between gene sets and biological signals in one dataset. It was conducted to identify the possible pathological processes of pSS. The top three significantly enriched gene sets were cytokine signaling in immune system, interferon signaling, and interferon alpha beta signaling in GSE66795 (Figure 2(e) and Table 3). Considering that the adjusted *P* value was greater than 0.05 in GSE84844, the enriched gene sets were not significant in this dataset (Table 3, adjusted *P* = 0.055).

### 3.3. PPI Network Analysis, MCODE Cluster Modules, and Hub Gene Identification

**Figure 3:**
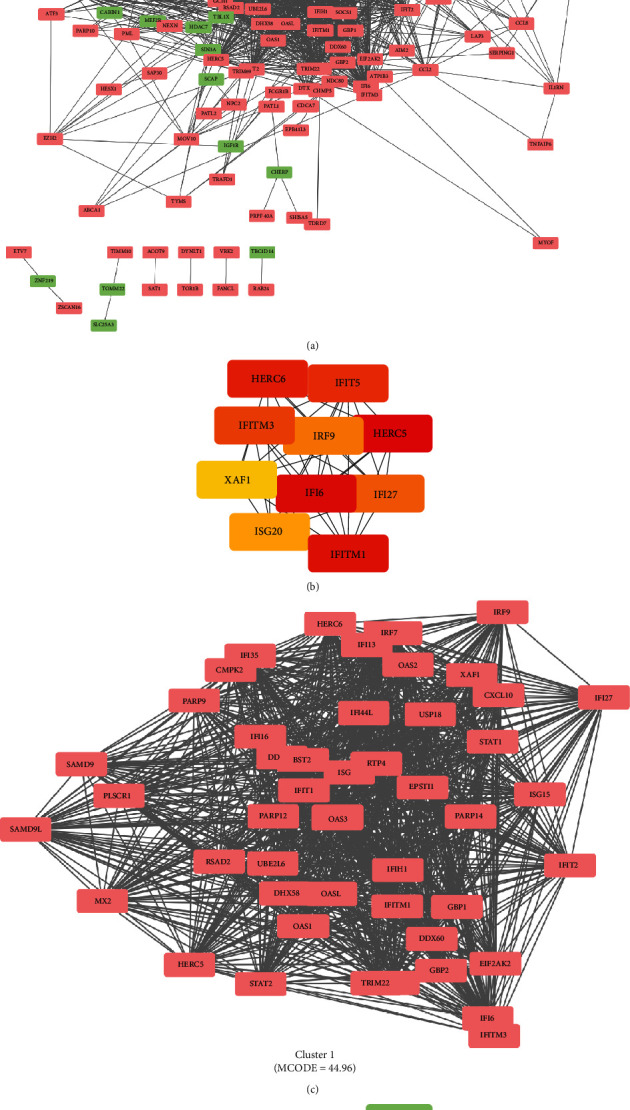
PPI network of DEGs and three cluster modules extracted by MCODE. (a) The interaction network between proteins coded by DEGs. (b) The interaction between the ten identified hub genes. Each node represents a protein, whereas each edge represents one protein–protein association. The purple rectangles represent the upregulated gene, and the yellow ones represent the downregulated gene. Two cluster modules extracted by MCODE. (c) Cluster 1 had higher cluster score (MCODE score = 44.96), followed by (d) cluster 2 (MCODE score = 4).

The PPI network for the 221 DEGs was constructed after the common DEGs were imported into STRING (Figure 3(a)). The top 10 hub genes were identified among the DEGs based on the information in the STRING database and Cytohubba. The top 10 hub genes were detected, including E3 ISG15-protein ligase HERC5, interferon alpha-inducible protein 6, interferon-induced transmembrane protein 1, probable E3 ubiquitin-protein ligase HERC6, interferon-induced protein with tetratricopeptide repeats 5, interferon-induced transmembrane protein 3, interferon alpha-inducible protein 27, interferon regulatory factor 9 (IRF9), interferon-stimulated gene 20 kDa protein, and XIAP-associated factor 1 (XAF1, Figure 3(b)). All the 10 hub genes were upregulated. The significant modules were identified by MCODE with an MCODE score of ≥4 as the threshold. Two modules with MCODE scores ≥ 4 are displayed in Figures 3(c) and 3(d). Cluster 1 (MCODE score = 44.96) had 51 nodes and 1124 edges (Figure 3(c)). Cluster 2 (MCODE score = 4) had 4 nodes and 6 edges (Figure 3(d)).

### 3.4. Screening and Verification of Diagnostic Markers

**Figure 4:**
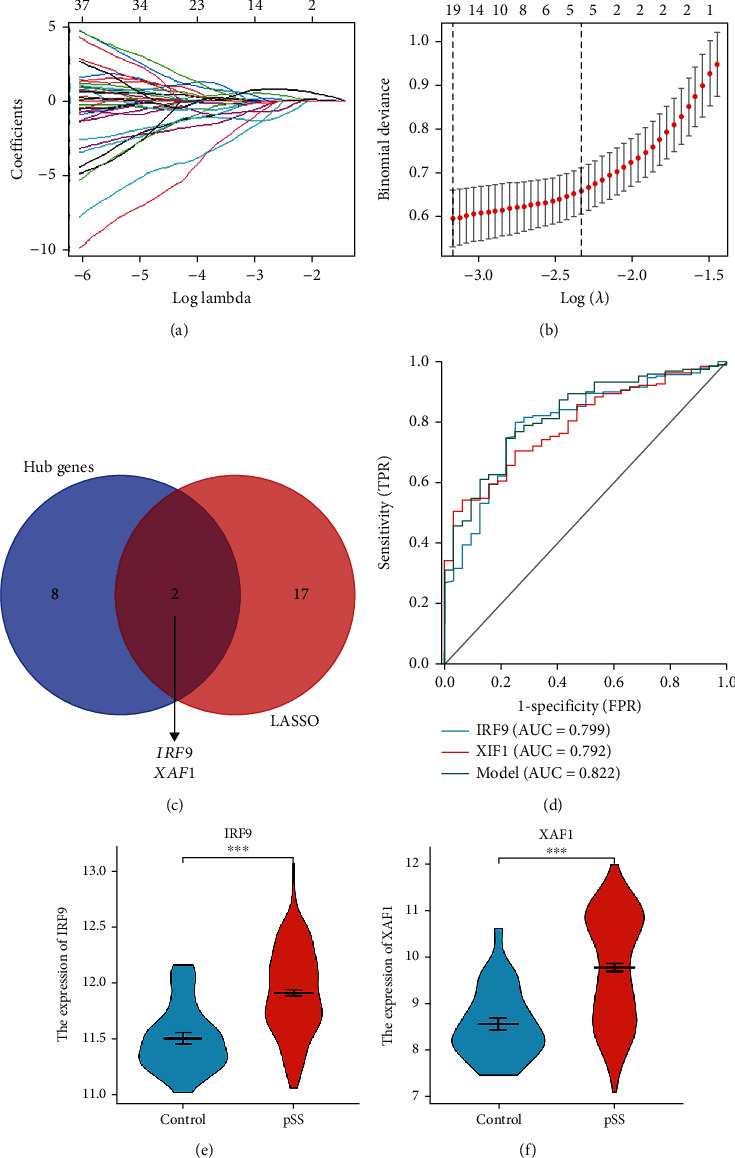
Screening and verification of diagnostic markers. (a) Least absolute shrinkage and selection operator (LASSO) logistic regression algorithm to screen diagnostic markers. Different colors represent different genes. Each curve corresponds to an independent variable in the full model prior to optimization. Curves indicate the path of each variable coefficient as *λ* varies. Lambda.min corresponds to *λ* which minimizes mean squared error in the model and was used for the selection of the seven predictor variables. (b) LASSO coefficient profiles of the 19 candidates in GSE66795. Plot of nonzero variable fit after cross-validation. Representation of the 10-fold cross-validation performed in LASSO that chooses the optimal *λ*. Lambda.min corresponds to *λ* which minimizes mean squared error and was used for variable selection. Lambda.1se corresponds to *λ* that is one standard error from Lambda.min. (c) Venn diagram shows the intersection of diagnostic markers obtained by LASSO and hub genes. (d) The ROC curve of the diagnostic efficacy tested by GSE51092. (e) The expression level of IRF9 between patients with pSS and HCs in GSE51092. (f) The expression level of XAF1 between patients with pSS and HCs in GSE51092. ^∗∗∗^*P* < 0.001.

The LASSO logistic regression algorithm identified 19 genes from the DEGs as diagnostic markers for pSS (Figures 4(a) and 4(b)). The gene markers obtained by LASSO algorithms and the hub genes overlapped. Finally, two diagnosis-related genes were obtained (Figure 4(c)). GSE51092 was used to validate the diagnostic efficacy of IRF9 and XAF1. The AUCs of IRF9 and XAF1 were 0.799 and 0.792, respectively, which indicated that IRF9 and XAF1 had certain diagnostic values (Figure 4(d)). When IRF9 and XAF1 were fitted into one variable, the diagnostic efficiency reached a higher level in the validation set (AUC = 0.822, Figure 4(d)). The identified logistic regression model was −27.6726 + (2.0467 × IRF9) + (0.6087 × XIF1). In addition, the GSE51092 dataset was used to verify the expression of the two identified genes. The mRNA expression levels of IRF9 and XAF1 were significantly increased in the pSS samples compared with the HCs (*P* < 0.01, Figures 4(e) and 4(f)).

## 4. Discussion

Although pSS is among the intractable autoimmune diseases, the number of studies focusing on the identification of important genes and pathways associated with pSS is far less than that on systemic lupus erythematosus and rheumatoid arthritis. The molecular and cellular events that occur during the pathogenesis of pSS need to be characterized. As the treatment of patients with pSS is still a clinical challenge, identifying the susceptibility genes of pSS is essential in studying the cause of pSS and find potential treatments.

In the present study, datasets containing the sequencing information of whole blood from pSS and HC were downloaded from the GEO database, and 221 DEGs were identified. The biological functions of these common DEGs were investigated. Defense response to virus, response to virus, and type I interferon signaling pathway were considerably enriched in pSS samples. In addition, the pathways enriched by GSEA involve cytokine signaling in immune system, interferon signaling, and interferon alpha beta signaling. The interferon pathway is activated in the pathogenesis of pSS as shown in previous research [21]. The first indication of interferon activation in pSS dated back to the late 1970s. In 2019, one study reported that salivary gland epithelial cells show upregulated interferon signaling pathway according to RNA-sequencing analysis [21]. Beyond salivary gland tissues, the presence of interferon signature was also evaluated at systemic level. The increased expression of interferon signaling pathway-associated genes or proteins has been detected in whole blood [22, 23], which is consistent with our findings. Interferons are mediators of innate immune defense mechanisms. The activation of innate immune pathways is a central pathogenetic contributor to pSS. Therefore, interferon blockade seems to be an attractive therapeutic target for this disease.

LASSO logistic regression was first proposed by Robert Tibshirani in 1996 [24]. It is a compressed estimation that determines the variable by finding *λ* when the classification error is the smallest. This algorithm is used to screen feature variables and build the best classification model. In this study, two diagnostic markers, namely, IRF9 and XAF1, were obtained by combining the genes identified by the LASSO logistic regression and the hub genes identified by Cytohubba. A hub gene plays a critical role in biological processes and is often influenced by the regulation of other genes in related pathways. Therefore, these two identified genes may play important roles in the pathogenesis of pSS and have good diagnostic performance. The expression level and diagnostic performance of IRF9 and XAF1 were tested by another gene dataset, GSE51092. IRF9 and XAF1 showed certain diagnostic accuracy with AUCs of 0.799 and 0.792, respectively. Moreover, the AUC of the combination of the two genes reached 0.822, indicating a good diagnostic performance. The identified logistic regression model was −27.6726 + (2.0467 × IRF9) + (0.6087 × XIF1). This model would be of great value in future clinical practice to diagnose pSS. IRF9 is an important transcription factor involved in type I interferon production [25]. The elevated level of IRF9 in patients with pSS has been reported in the past [26]. Its binding with the signal transducer and activator of transcription 1 and 2 heterodimer leads to the formation of a complex, namely, interferon-stimulated gene factor (ISG) 3 [27]. The encoded ISG proteins have several immunomodulatory functions, including the induction of B cell activating factor, immunoglobulin switching, and increased antigen presentation, which all have vital roles in the pathogenesis of pSS [28, 29]. XAF1, an interferon type I inducible gene, encodes a zinc-finger proapoptotic protein. The role of XAF1 is discussed in different kinds of cancers. XAF1 works as a modifier of p53 function and cancer susceptibility [30]. Limited studies focused on the role of XAF1 in pSS. Two studies reported its elevation in patients with pSS [7, 31]. Our result showed that IRF9 and XAF1 had certain diagnostic abilities. Therefore, future precisely designed studies are necessary to verify these potential genes.

In the interpretation of our results, the following limitations require careful discussion. On the one hand, the clinical samples included in this study were from different datasets. The clinical activity, severity, or gender may be different among groups. Therefore, heterogeneity and confounding factors may have distorted the analysis. On the other hand, our study is only a bioinformatic study, and the identified hub genes were not confirmed through in vitro assays or in vivo models. Hence, further precisely designed studies are necessary to verify the two identified genes.

In conclusion, the biological analyses provided an overview of the differential gene expression between pSS and HC, which determined 221 DEGs. The functional analysis of these DEGs indicated that defense response to virus, response to virus, and type I interferon signaling pathway were considerably enriched in the whole blood of patients with pSS. In addition, two diagnostic markers, IRF9 and XAF1, were obtained and proven to have certain diagnostic accuracy. Our analysis revealed previously unknown transcriptional changes in pSS and demonstrated the role of microarray-based expression profiling in characterizing biomarkers in diseases. However, the exact diagnostic values of the identified models still need to be tested in future large-scale investigations. The results of our study may provide new treatment targets for pSS. Hence, this analysis may guide future experimental research and clinical transformation.

## Figures and Tables

**Table 1:** Information for selected microarray datasets.

GEO accession	Platform	Samples		Source tissue	Attribution
		pSS	HC		
GSE84844	GPL570	30	30	Whole blood	Test set
GSE66795	GPL10558	131	29	Whole blood	Test set
GSE51092	GPL6884	190	32	Whole blood	Validation set

pSS: primary Sjogren syndrome; HC: healthy control.

**Table 2:** GO and KEGG analyses of DEGs.

Ontology	ID	Description	Gene ratio	Adjusted *P*	*q* value
BP	GO:0051607	Defense response to virus	46/199	1.40*e* − 41	1.29*e* − 41
BP	GO:0009615	Response to virus	49/199	2.82*e* − 39	2.59*e* − 39
BP	GO:0060337	Type I interferon signaling pathway	28/199	2.78*e* − 30	2.55*e* − 30
MF	GO:0003725	Double-stranded RNA binding	10/201	5.37*e* − 06	4.91*e* − 06
MF	GO:0003950	NAD+ ADP-ribosyltransferase activity	4/201	0.015	0.014
MF	GO:0003727	Single-stranded RNA binding	7/201	0.015	0.014
KEGG	hsa05164	Influenza A	21/117	5.07*e* − 12	4.82*e* − 12
KEGG	hsa04621	NOD-like receptor signaling pathway	16/117	5.37*e* − 07	5.10*e* − 07

CC: cellular component group; MF: molecular function group; BP: biological process group; KEGG: Kyoto Encyclopedia of Genes and Genomes.

**Table 3:** Gene set enrichment analysis.

ID	NES	Adjusted *P*	FDR
GSE84844			
Reactome cytokine signaling in immune system	1.614	0.055	0.051
Reactome translation	1.74	0.055	0.051
Reactome interferon signaling	2.468	0.055	0.051
Reactome regulation of expression of SLITs and ROBOs	1.883	0.055	0.051
Reactome influenza infection	2.016	0.055	0.051
GSE66795			
Reactome cytokine signaling in immune system	2.085	0.01^∗∗∗^	0.007
Reactome interferon signaling	2.185	0.01^∗∗∗^	0.007
Reactome interferon alpha beta signaling	2.18	0.01^∗∗∗^	0.007
Reactome antiviral mechanism by IFN-stimulated genes	2.077	0.01^∗∗∗^	0.007
WP host pathogen interaction of human coronaviruses' interferon induction	1.777	0.025^∗^	0.018

^∗∗∗^Adjusted *P* ≤ 0.01; ^∗^adjusted *P* ≤ 0.05.

## Data Availability

The datasets generated and/or analyzed during the current study are available in the GEO repository. GEO is a free, publicly available repository database that stores a large number of gene functions and expression. The working links are as follows: GSE84844 (https://www.ncbi.nlm.nih.gov/geo/query/acc.cgi?acc=GSE84844), GSE66795 (https://www.ncbi.nlm.nih.gov/geo/query/acc.cgi?acc=GSE66795), and GSE51092 (https://www.ncbi.nlm.nih.gov/geo/query/acc.cgi?acc=GSE51092).

## Supplementary Materials

Supplementary Materials Supplementary Figure 1: a flowchart of the study.
