# Supplementary material for: IRF9 and XAF1 as Diagnostic Markers of Primary Sjogren Syndrome
Source: Comput Math Methods Med. 2022 Sep 12;2022:1867321. doi: 10.1155/2022/1867321 (PMC9484944; doi:10.1155/2022/1867321)
Supplement: Supplementary Materials — Supplementary Figure 1: a flowchart of the study. [file 1867321.f1.docx]

GSE84844

(GPL570)

GSE66795

(GPL10558)

Identification of DEGs

Functional and pathway (GO and KEGG) enrichment analysis

Construction of the PPI network, MCODE cluster modules

GSE51092 (GPL6884)

Verification of the identified hub genes

ROC curve of the hub genes

Test sets

Validation set

Identification of hub genes by Cytohubba

Identification of hub genes by LASSO

Overlapping hub genes
